# Correction: Direct synthesis of the organic and Ge free Al containing BOG zeolite (ITQ-47) and its application for transformation of biomass derived molecules

**DOI:** 10.1039/d0sc90248a

**Published:** 2020-11-05

**Authors:** Qintong Huang, Ningyue Chen, Lichen Liu, Karen S. Arias, Sara Iborra, Xianfeng Yi, Chao Ma, Weichi Liang, Anmin Zheng, Chuanqi Zhang, Jibo Hu, Zilin Cai, Yi Liu, Jiuxing Jiang, Avelino Corma

**Affiliations:** MOE Key Laboratory of Bioinorganic and Synthetic Chemistry, School of Chemistry, Sun Yat-sen University Guangzhou 510275 P. R. China jiangjiux@mail.sysu.edu.cn; Instituto de Tecnología Química, Universitat Politècnica de València-Consejo Superior de Investigaciones Científicas Avenida de los Naranjos s/n 46022 Valencia Spain acorma@itq.upv.es; State Key Laboratory of Magnetic Resonance and Atomic and Molecular Physics, National Center for Magnetic Resonance in Wuhan, Wuhan Institute of Physics and Mathematics, Innovation Academy for Precision Measurement Science and Technology, Chinese Academy of Sciences Wuhan 430071 P. R. China; Zhang Dayu School of Chemistry, Dalian University of Technology Dalian 116024 Liaoning China; ZhongShan AKDM Biological Technology Co., Ltd Zhongshan 528400 P. R. China; School of Materials Science and Engineering, Zhengzhou University Zhengzhou Henan 450001 P. R. China

## Abstract

Correction for ‘Direct synthesis of the organic and Ge free Al containing BOG zeolite (ITQ-47) and its application for transformation of biomass derived molecules’ by Qintong Huang *et al.*, *Chem. Sci.*, 2020, DOI: 10.1039/d0sc04044d.

In the original manuscript, the affiliations of authors Anmin Zheng and Avelino Corma were displayed incorrectly. The corrected affiliations are as displayed above.

The Royal Society of Chemistry apologises for these errors and any consequent inconvenience to authors and readers.

